# Diagnostic and Therapeutic Challenges in Concurrent Addison’s Disease and Acromegaly

**DOI:** 10.7759/cureus.88323

**Published:** 2025-07-19

**Authors:** Stylianos Kopanos, Joachim Feldkamp

**Affiliations:** 1 Academic Department of Endocrinology, Diabetes and Infectiology, Klinikum Bielefeld, Medical School and University Medical Centre East Westphalia-Lippe Bielefeld University, Bielefeld, DEU

**Keywords:** acromegaly, addison’s disease, autoimmune polyendocrine syndrome, endocrine disorders, pituitary adenoma, transsphenoidal surgery

## Abstract

The simultaneous occurrence of Addison’s disease and acromegaly presents a unique and complex clinical challenge. Addison’s disease, characterized by autoimmune adrenal destruction, results in cortisol and aldosterone deficiencies, while acromegaly stems from excessive growth hormone secretion, usually due to a pituitary adenoma. Their coexistence complicates diagnosis and management due to overlapping systemic effects.

We report a 53-year-old woman initially diagnosed with Addison’s disease, presenting with fatigue, hypotension, and hyperpigmentation. Four years later, she developed acromegalic symptoms, including acral enlargement and hyperhidrosis. Laboratory assessments confirmed elevated insulin-like growth factor-1 (IGF-1) and growth hormone levels, with imaging revealing two pituitary microadenomas. The patient underwent transsphenoidal surgery, achieving tumor debulking and hormonal stabilization. Addison’s disease management involved hydrocortisone, fludrocortisone, and levothyroxine, complicated by metabolic instability and hypertension. Additional comorbidities included diabetes, glaucoma, and sleep apnea, necessitating a multidisciplinary approach. Long-term follow-up demonstrated stable IGF-1 levels and no recurrence.

This case illustrates the rare co-occurrence of Addison’s disease and acromegaly, highlighting diagnostic delays, difficulties in endocrine management, and the critical importance of structured long-term follow-up. Special attention is paid to the impact of incomplete mineralocorticoid evaluation, missed opportunities for early diabetes screening, and evolving pituitary insufficiency after tumor resection.

## Introduction

Addison’s disease, a rare and potentially fatal disorder, arises from inadequate adrenal hormone production. It results from primary adrenal cortex damage or secondary/tertiary causes like insufficient adrenocorticotropic hormone (ACTH) or corticotropin-releasing hormone (CRH) secretion. Diagnosis, often delayed, relies on identifying low cortisol levels, with many patients first presenting in adrenal crises. In Western populations, autoimmune adrenalitis is the most common cause, responsible for 70%-85% of primary adrenal insufficiency (PAI) cases [1]. Other causes include infections (e.g., tuberculosis), adrenal hemorrhage, and genetic mutations (e.g., CYP21A2). PAI symptoms such as fatigue, weight loss, nausea, and hyperpigmentation often overlap with other conditions, complicating diagnosis. Management involves lifelong steroid replacement and, in tertiary cases, gradual tapering of glucocorticoids [2].

Acromegaly, a rare disorder caused by excessive growth hormone (GH) secretion, typically results from benign pituitary adenomas. Chronic GH and insulin-like growth factor-1 (IGF-1) overproduction cause systemic complications such as hypertension, diabetes, and cardiomyopathy [3,4]. Characteristic features include acral enlargement, coarsened facial features, and hyperhidrosis. Management combines surgery, medical therapy, and sometimes radiotherapy, aiming to normalize GH/IGF-1 levels and mitigate complications [5,6].

Addison’s disease, a rare disorder of PAI, is most commonly autoimmune in origin in Western populations. Acromegaly, in contrast, is a rare endocrine disorder caused by chronic hypersecretion of GH, typically from a pituitary adenoma. While both diseases are uncommon on their own, their coexistence in a single patient is exceedingly rare, and to our knowledge, very few documented cases exist in the literature. The mechanisms underlying such coexistence are not well understood but may include chance association, shared autoimmune predisposition, or dysregulation of the hypothalamic-pituitary-adrenal (HPA) axis.

Our case highlights a 53-year-old woman with a history of thyroid surgery and evolving symptoms initially attributed to adrenal insufficiency, who later developed clinical and biochemical signs of acromegaly. This case illustrates the diagnostic complexity and management challenges of overlapping endocrinopathies and underscores the importance of long-term endocrine surveillance, particularly in patients with evolving or unexplained systemic symptoms.

## Case presentation

A 53-year-old Caucasian woman was initially referred to our internal medicine clinic with a history of 5 kg weight loss, abdominal discomfort, joint and muscle pain, constipation, fatigue, lethargy, orthostatic hypotension (systolic arterial pressure (SAP) 75-80 mmHg), dizziness, and nausea. She also exhibited skin hyperpigmentation.

Her medical history included post-surgical hypothyroidism following goiter resection three years prior, managed with levothyroxine, with no evidence of autoimmunity. Cardiological evaluations showed no abnormalities, including bradyarrhythmia, branch blocks, or other electrocardiographic changes. Recent endoscopic examinations revealed no gastrointestinal pathology. Additional symptoms included confusion, libido loss, anxiety, edema, and vertigo.

The patient’s only medication was levothyroxine. Both physical and psychomotor examinations were unremarkable, and there was no family history of hyponatremia or endocrinological disorders. The patient was an ex-smoker (ca. 35 pack-years).

Diagnostic assessment

Initial laboratory evaluation revealed significant hyponatremia (serum sodium 120 mmol/L), low urinary sodium excretion, and markedly reduced morning cortisol levels (2.5 μg/L), all supporting the diagnosis of PAI. The patient’s gonadotrope axis showed elevated follicle-stimulating hormone (FSH) and luteinizing hormone (LH) levels, consistent with postmenopausal status. Although estradiol was measurable at 157 pg/mL, this isolated elevation was not reproducible and was not accompanied by clinical signs of estrogen excess, suggesting possible assay variability or transient fluctuation. Further hormonal assessment showed no signs of generalized hypopituitarism. Thyroid function tests presented no pathological concentrations. Regarding autoimmune markers, parietal antibodies were positive at 1:80 (reference range: <1:10) (Table 1).

**Table 1:** Laboratory and endocrine data at first visit. DHEAS: dehydroepiandrosterone sulfate; ACTH: adrenocorticotropic hormone; PTH: parathyroid hormone; LH: luteinizing hormone; FSH: follicle-stimulating hormone; HGH: human growth hormone; TSH: thyroid-stimulating hormone; fT3: free triiodothyronine; fT4: free thyroxine; TPO antibodies: thyroid peroxidase antibodies; ADR antibodies: adrenal antibodies

Parameters	Result	Reference range
Sodium	120 mmol/L	135–145 mmol/L
24-hour urine sodium	73 mmol/24 h	100–220 mmol/24 h
Cortisol	2.5 μg/L	5–25 μg/L
DHEAS	0 μg/dL	35–430 μg/dL
ACTH	1,489 pg/mL	10–50 pg/mL
Phosphorus	3.0 mg/dL	2.7–4.5 mg/dL
Calcium	2.4 mmol/L	2.2–2.5 mmol/L
Potassium	4.2 mmol/L	3.5–5.2 mmol/L
Glucose	112 mg/dL	80–100 mg/dL
PTH	58.8 pg/mL	15–65 pg/mL
LH	51.6 mE/mL	Not provided
FSH	32.1 mE/mL	Not provided
Estradiol	157 pg/mL	Not provided
Testosterone	2.14 nmol/L	0.5–2.4 nmol/L
Prolactin	8.6 ng/mL	<25 ng/mL
HGH	1.3 ng/mL	<5 ng/mL
TSH	0.37 μE/mL	0.27–4.2 μE/mL
fT3	1.4 pg/mL	2.0–4.4 pg/mL
fT4	1.2 ng/mL	0.9–1.7 ng/mL
TPO antibodies	11 U/L	≤35 U/L
Thyroglobulin antibodies	24 U/L	≤115 U/L
Parietal antibodies	1:80	<1:10
Hemoglobin	13.4 g/dL	12.0–16.0 g/dL
ADR antibodies	Negative	<10 U/L

**Figure 1:**
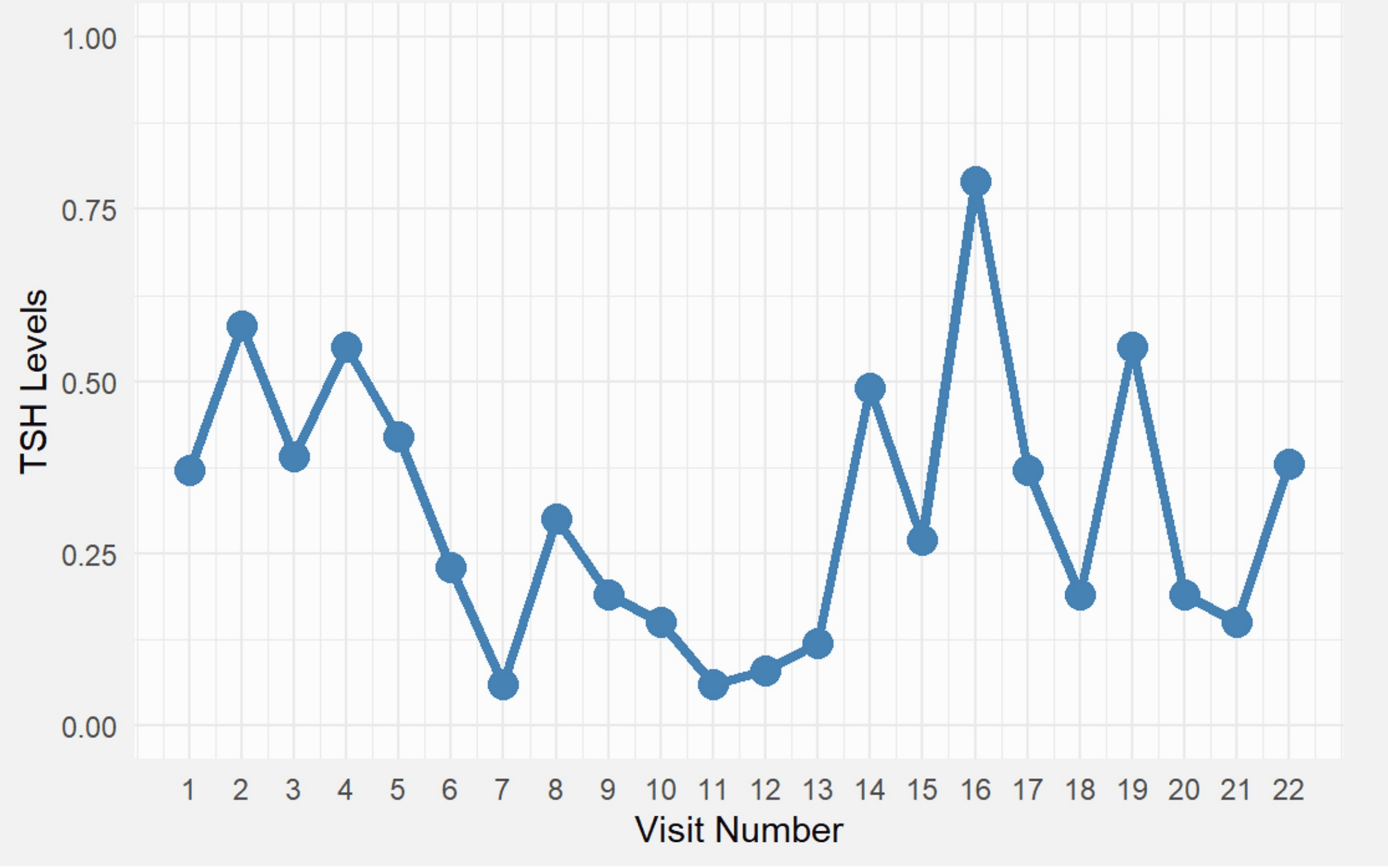
Serum thyroid-stimulating hormone (TSH) levels across 22 outpatient visits. TSH fluctuated between 0.05 and 0.80 μU/mL, reflecting evolving thyroid axis activity and prompting levothyroxine dose adjustments (increased from 100 to 115 μg/day). Visit numbers correspond to follow-up milestones post-adrenal insufficiency diagnosis and post-pituitary surgery. TSH levels fluctuated mildly throughout the follow-up, ranging from 0.42 to 0.84 μU/mL, under levothyroxine replacement. At visit 10, a dose adjustment from 100 to 115 μg/day was made in response to fatigue and borderline low fT4 levels. These changes were temporally associated with subtle shifts in TSH, but remained within normal limits throughout.

A neck ultrasound demonstrated the absence of the right thyroid lobe post-thyroidectomy, while the left thyroid lobe had a volume of approximately 3.5 mL and showed inhomogeneous echotexture without features of autoimmune thyroiditis. Abdominal ultrasound identified the presence of liver cysts but revealed no evidence of malignancy within the abdominal cavity. Cerebral magnetic resonance imaging (MRI) showed no findings consistent with an empty sella or the presence of a pituitary adenoma (Figure 1).

**Figure 2:**
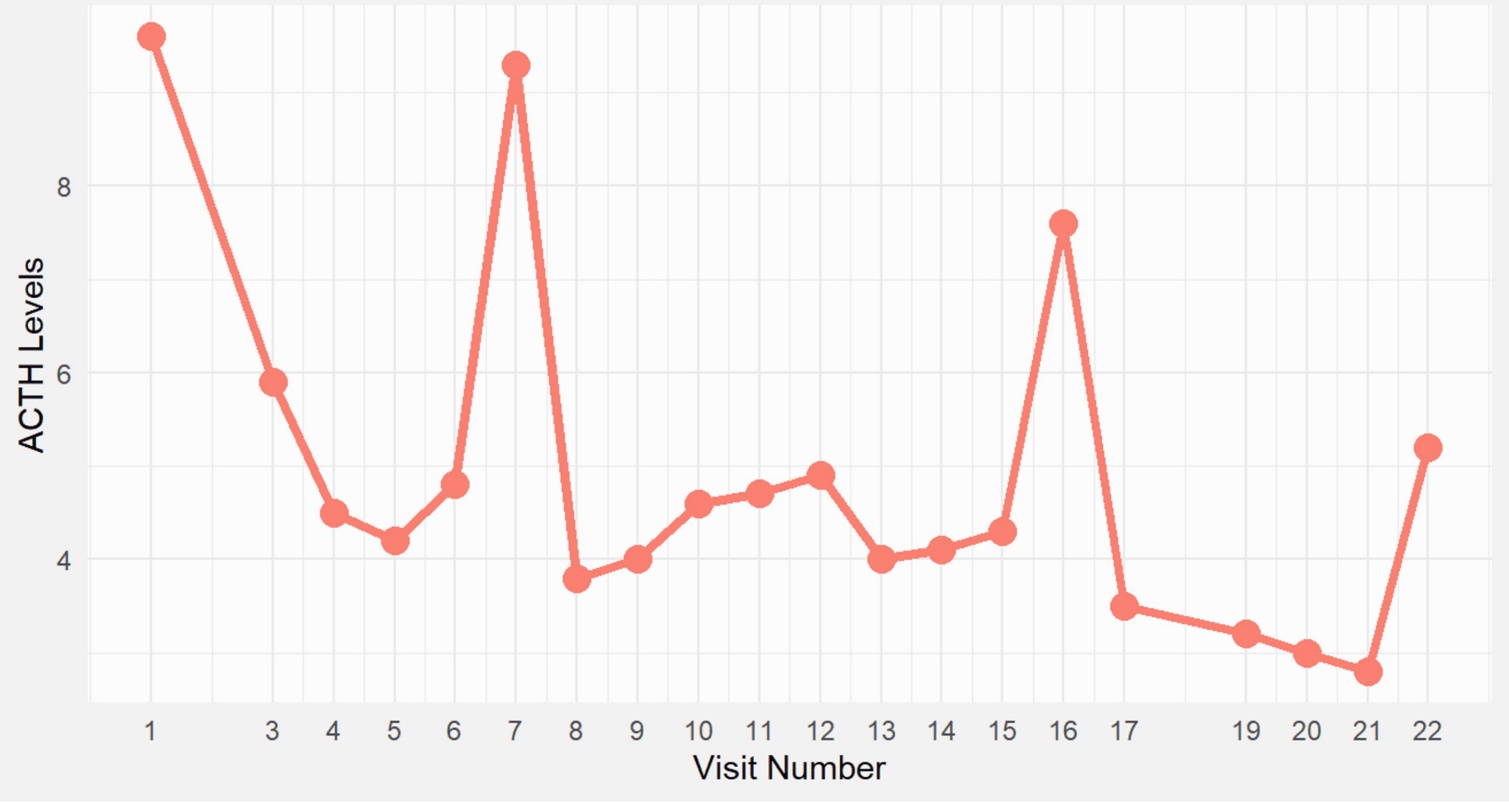
Plasma adrenocorticotropic hormone (ACTH) levels during follow-up. ACTH ranged from 3.5 to 9.5 pg/mL after initiation of hydrocortisone therapy for Addison’s disease. Peaks represent inconsistent adherence or stress-induced activation. Despite fluctuations, ACTH remained elevated, consistent with primary adrenal insufficiency. Notably, ACTH levels peaked around visit 6, coinciding with increased fatigue and borderline low cortisol levels. The hydrocortisone dose was increased to 25 mg/day shortly after this point, leading to partial symptom relief, though ACTH remained high.

**Figure 3:**
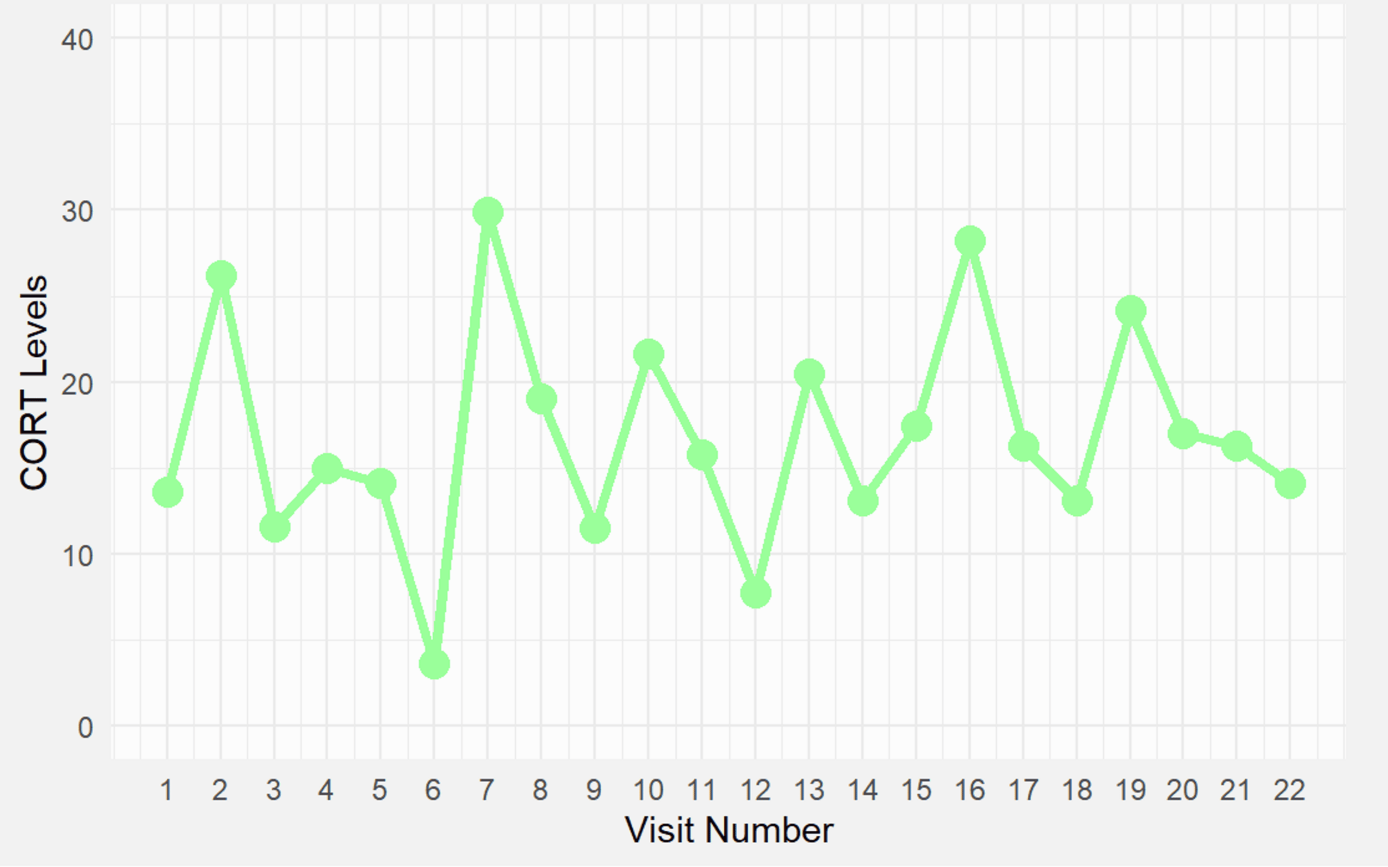
Serum cortisol (CORT) levels over 22 outpatient visits. Cortisol levels fluctuated between 6 and 32 μg/dL during the follow-up of primary adrenal insufficiency. Measurements reflect variable timing relative to hydrocortisone intake, which complicates interpretation. These results were used to guide dose adjustments (hydrocortisone 15–25 mg/day) based on symptoms such as fatigue, blood pressure instability, and stress exposure. Cortisol levels ranged from 6 to 32 μg/dL and fluctuated depending on the timing of sampling relative to hydrocortisone dosing. The increase in cortisol levels after visit 7 coincided with a hydrocortisone dose adjustment and the addition of fludrocortisone. Consistent cortisol timing documentation was introduced later in the course to improve monitoring accuracy.

Due to weight gain and a mild hypothyroid state, levothyroxine therapy was maintained at a dosage of 100 μg/day. Adrenal insufficiency was confirmed based on low morning cortisol and elevated ACTH (Figures 2, 3). Given the clinical urgency, hydrocortisone and fludrocortisone therapy were initiated based on hypotension and hyponatremia, even though mineralocorticoid markers (renin, potassium) were incomplete. Autoimmune adrenalitis was suspected. Substitution therapy with hydrocortisone (25 mg/day) and fludrocortisone (0.5 mg/day) was initiated to address hormonal deficiencies, aiming to replicate the natural diurnal cortisol rhythm and support metabolic regulation, immune function, and stress adaptation. The patient received comprehensive education on maintaining hormonal balance, preventing adrenal crises, and avoiding complications associated with both under-replacement and over-replacement of therapy.

Over the next four years of follow-up in our endocrinology outpatient clinic, the patient experienced fluctuations related to over- or underdosing of substitution therapy, including paroxysmal tachycardias, cognitive impairments in concentration, and changes in weight and endurance. Neck sonography identified recurrent goiter in the left thyroid gland. Serial evaluations showed fluctuating hormone levels: thyroid-stimulating hormone (TSH) ranged from 0.42 to 0.84 μU/mL, IGF-1 decreased from 495 to 240 ng/mL, and 24-hour urinary cortisol ranged from 64 to 108 μg/24 h. These changes paralleled clinical symptoms such as weight changes, tachycardia, and fatigue. In response, levothyroxine was increased from 100 to 115 μg/day, hydrocortisone was adjusted between 15 and 25 mg/day, and fludrocortisone was given 3-5 times per week (0.5 mg each) based on blood pressure, electrolytes, and fatigue levels. Ergometry results were satisfactory, with no electrocardiographic abnormalities observed. Echocardiography revealed no evidence of structural cardiomyopathy or valvular insufficiency. No clinical manifestations of autoimmune polyglandular syndrome (candidiasis, alopecia, or vitiligo) were exhibited.

**Figure 4:**
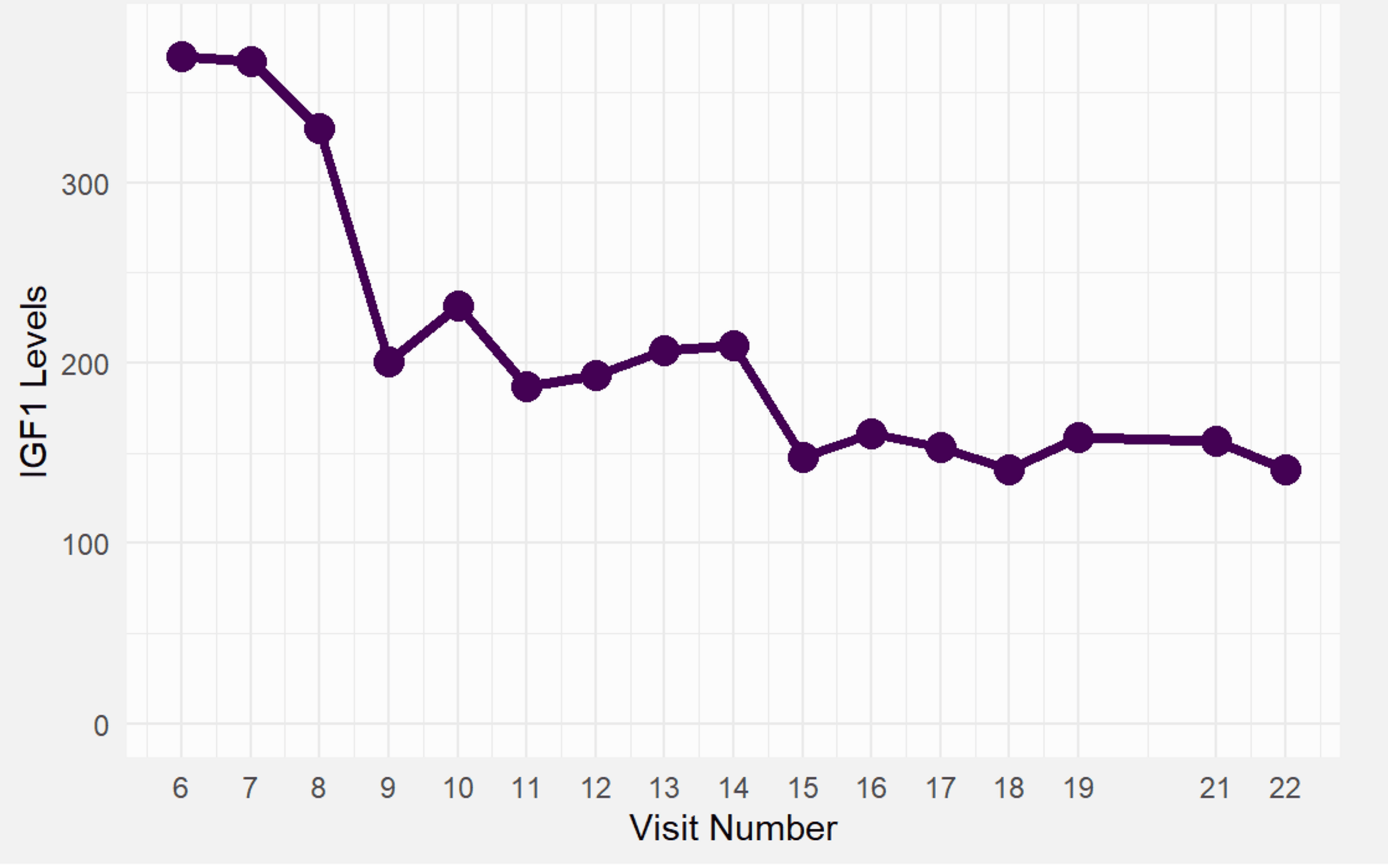
Insulin-like growth factor-1 (IGF-1) levels from diagnosis of acromegaly through post-operative follow-up. Levels declined from >370 ng/mL at diagnosis to ~160 ng/mL post-transsphenoidal surgery, demonstrating hormonal control without the need for somatostatin analogues. IGF-1 levels correlated with symptom improvement (reduction in sweating, sleep apnea, and acral changes). IGF-1 levels peaked at visit 14, during stable adrenal and thyroid replacement, and before surgery. Following transsphenoidal resection (visit 15), IGF-1 declined by over 50%.

In the fifth year of follow-up, the patient presented with worsening nervousness, sleep disturbances, palpitations, hyperhidrosis, headaches, and skin tags. Clinical reevaluation revealed coarse facial features, large hands, and a history of untreated snoring. Hormonal evaluation indicated a shift toward biochemical hyperthyroidism, with TSH at 0.06 μU/mL and elevated free triiodothyronine (fT3) at 4.1 pg/mL and free thyroxine (fT4) at 2.4 ng/mL. This likely reflected a transient oversubstitution with levothyroxine, as no sonographic or scintigraphic evidence of autonomous thyroid function or thyroiditis was identified. IGF-1 was significantly elevated at 367 ng/mL and confirmed by a GH stimulation test showing IGF-1 levels of 495 ng/mL (Figure 4). Although the oral glucose tolerance test (OGTT) is the standard confirmatory test for acromegaly, it was not performed due to the risk of hypoglycemia in the early phase of hydrocortisone replacement in a patient with untreated adrenal insufficiency. While glucocorticoids can induce hyperglycemia chronically, this patient had not yet achieved metabolic stability, and cortisol deficiency itself can predispose to fasting hypoglycemia; thus, due to concern for potential hypoglycemic reactions during glucose loading, OGTT testing was avoided. In this early phase of treatment, cortisol regulation was not yet stable, and given cortisol’s key role in gluconeogenesis and counter-regulatory response, the risk of symptomatic hypoglycemia was deemed significant. Diagnosis was based on markedly elevated IGF-1 levels (367-495 ng/mL), consistent clinical features, and MRI findings of pituitary microadenomas.

The patient underwent successful transsphenoidal resection of two GH-secreting microadenomas. Postoperatively, IGF-1 levels declined from 495 to 240 ng/mL, and no residual tumor tissue was identified on follow-up MRI. Given the favorable biochemical response and absence of persistent symptoms, no adjuvant medical therapy (e.g., somatostatin analogues, cabergoline, or pegvisomant) was required. The patient remained in biochemical remission over two years of follow-up. This therapeutic approach was particularly influenced by her complex endocrine background, including Addison’s disease, hypothyroidism, and emerging hypopituitarism, necessitating careful hormonal surveillance rather than immediate pharmacologic escalation.

Visit numbers used in Figures 1-4 reflect consecutive endocrine clinic follow-ups from the time of initial diagnosis. Clinical milestones such as diagnosis, surgery, and treatment adjustments are detailed in the text. Notably, following visit 7, the patient reported worsening fatigue and orthostatic hypotension, prompting an increase in hydrocortisone to 25 mg/day and initiation of regular fludrocortisone at 0.5 mg/day. Visit 8 marked the beginning of symptom improvement, paralleled by a rise in serum cortisol levels (Figure 3), a slight rebound in ACTH (Figure 2), and later stabilization of TSH (Figure 1). Cortisol increased after visit 7, following adjustments to hydrocortisone and fludrocortisone doses due to clinical deterioration. The timing of sampling was inconsistent early in follow-up but improved later, aligning cortisol interpretation more accurately with therapy response.

**Figure 5:**
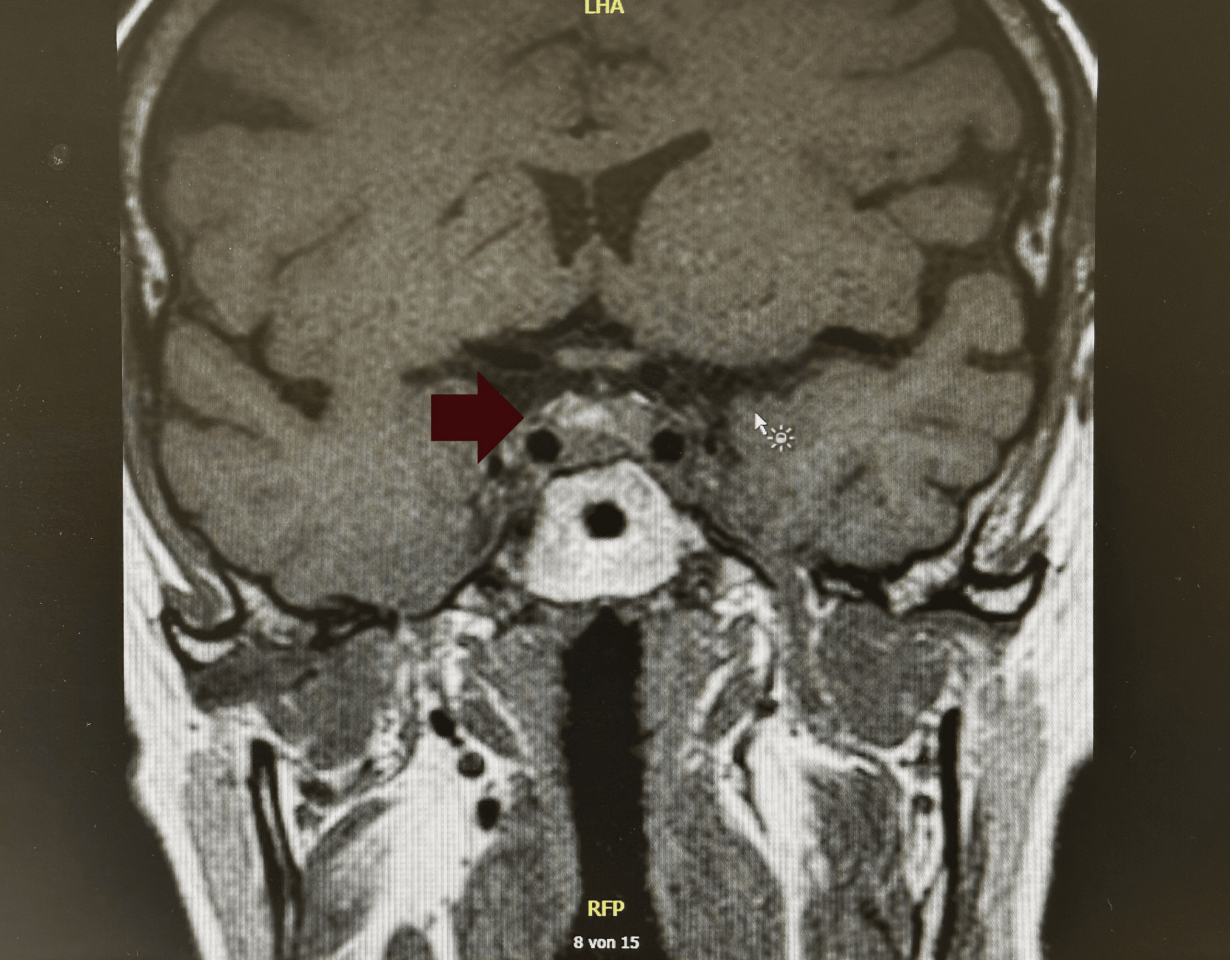
Coronal T1-weighted MRI showing a GH-secreting pituitary macroadenoma (10 × 14 × 9 mm) occupying the sella turcica (red arrow). Preoperative image showing the larger of two intrasellar lesions (red arrow) expanding the sella turcica, consistent with a macroadenoma. At the time of imaging, the patient’s ACTH level was markedly elevated (1,489 pg/mL), supporting a diagnosis of primary adrenal insufficiency (Addison’s disease) rather than pituitary dysfunction or compression. The tumor was later confirmed to be GH-secreting, not ACTH-secreting. MRI: magnetic resonance imaging; GH: growth hormone; ACTH: adrenocorticotropic hormone

**Figure 6:**
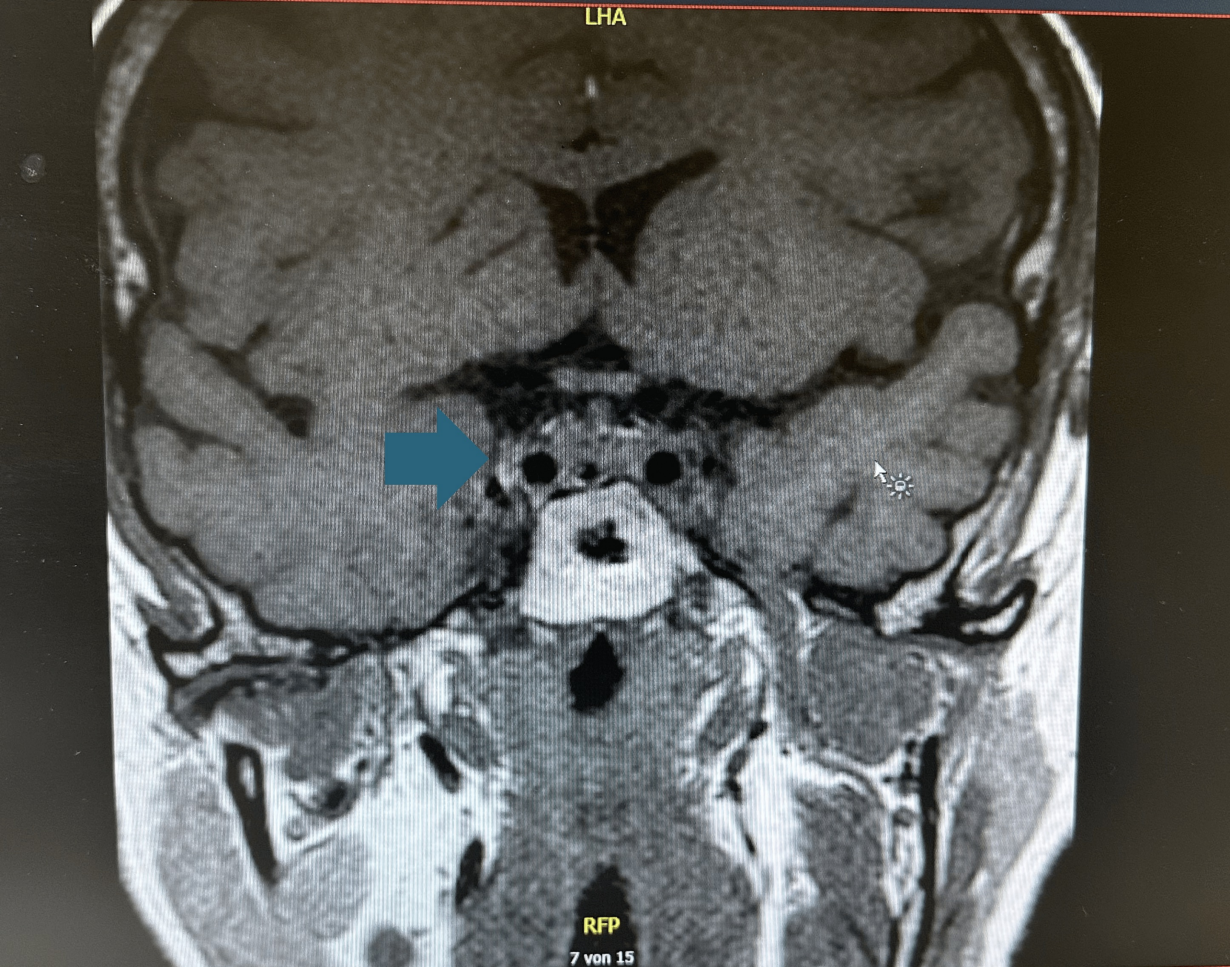
Postoperative coronal T1-weighted MRI of the pituitary region. The image shows post-debulking changes following transsphenoidal resection of a GH-secreting pituitary macroadenoma. The previously visualized intrasellar mass (see Figure 5) has been substantially reduced, with no compression of the optic chiasm or cavernous sinus. Residual soft tissue density (blue arrow) likely reflects postoperative changes or minimal residual tumor. At this time point, IGF-1 levels had declined significantly, and histopathology confirmed a densely granulated somatotroph adenoma with positive GH immunostaining and a Ki-67 index < 2%. MRI: magnetic resonance imaging; GH: growth hormone; IGF-1: insulin-like growth factor-1

Cerebral MRI revealed two pituitary microadenomas: one measuring 10 × 14 × 9 mm and another intrasellar lesion measuring 3 × 4 × 6 mm, in contact with the cavernous sinus and internal carotid artery, without elevation of the optic chiasm (Figures 5, 6). Thyroid scintigraphy excluded autonomous nodules and malignancy.

Histopathological examination confirmed a GH-secreting pituitary adenoma. Immunohistochemistry revealed strong positivity for GH, with negative staining for ACTH, prolactin (PRL), and TSH. The Ki-67 index was <2%, and mitotic figures were absent. The adenoma was identified as a densely granulated somatotroph tumor, supporting the clinical and biochemical diagnosis of acromegaly.

## Discussion

This case emphasizes the importance of recognizing overlapping endocrine disorders and the management challenges that arise. Addison’s disease symptoms can obscure acromegaly findings initially. Further, rapid hydrocortisone replacement may unmask latent pituitary insufficiency. Early diagnosis of acromegaly is essential to screen and manage associated comorbidities, including diabetes and cardiac disease. Clinical judgment sometimes requires treatment initiation before full laboratory confirmation, particularly in adrenal crises. However, structured re-evaluation is mandatory.

At the time of IGF-1 measurement (495 ng/mL), the patient had been on stable hydrocortisone and levothyroxine replacement for over four weeks, with resolution of adrenal crisis symptoms. Although OGTT was not performed due to hypoglycemia risk, the diagnosis of acromegaly was supported by sustained IGF-1 elevation, characteristic physical findings, a pituitary macroadenoma on MRI, and histopathological confirmation of GH secretion postoperatively. The combination of PAI, hypothyroidism, and positive parietal antibodies also points toward a multisystem autoimmune syndrome, likely Schmidt syndrome (autoimmune polyendocrine syndrome (APS) type 2), despite the absence of classic thyroid antibodies.

Although thyroid dysfunction was not the primary focus of this case, it significantly influenced the patient’s clinical course and diagnostic trajectory. The hypothyroidism resulted from prior partial thyroidectomy, with no evidence of autoimmune thyroid disease (negative thyroid peroxidase (TPO) and thyroglobulin (Tg) antibodies; non-specific ultrasound). While acromegaly may contribute to thyroid hyperplasia or goiter recurrence, it rarely causes functional hypothyroidism. Importantly, thyroid hormone fluctuations - especially during levothyroxine dose adjustments - overlapped with symptoms of adrenal insufficiency and acromegaly (e.g., fatigue, weight gain, and blood pressure instability), thereby confounding the early differential diagnosis. As such, the thyroid disorder functioned as a third, independent endocrinopathy that complicated both recognition and longitudinal management of the patient’s Addison’s disease and GH-secreting pituitary adenoma.

Acromegaly, caused by pituitary adenomas, and Addison’s disease, resulting from adrenal insufficiency, are rare conditions with an extremely low likelihood of co-occurrence. Addison’s is often autoimmune, while acromegaly typically lacks autoimmunity, but shared mechanisms, such as genetic predispositions (e.g., HLA or GNAS mutations) or HPA axis dysregulation, could link these disorders [7]. For example, cortisol deficiency in Addison’s may trigger compensatory pituitary changes, while excess GH and IGF-1 in acromegaly can dysregulate adrenal function, exacerbating hormonal imbalances [8,9].

Both disorders are associated with significant metabolic and cardiovascular complications, including hypertension, glucose intolerance, and cardiomyopathy [10]. Chronic inflammation, oxidative stress, and vascular changes in acromegaly may further aggravate Addison’s-related hypotension. Managing such overlapping comorbidities requires a multidisciplinary approach [11-13].

Diagnosing these coexisting conditions is challenging due to overlapping symptoms and potential lab test confounders, such as the effect of hypocortisolism on OGTT and IGF-1 levels. While elevated IGF-1 is a hallmark of acromegaly, it may also be elevated in non-pituitary conditions such as poorly controlled diabetes mellitus, hepatic dysfunction, or renourishment states. In our patient, although diabetes was later diagnosed, hemoglobin A1c (HbA1c) was only mildly elevated (6.3%-7.2%), and there was no evidence of hepatic or nutritional abnormalities. The presence of classic acromegalic features and a pituitary macroadenoma, along with histological confirmation of a GH-secreting adenoma, helped definitively establish the diagnosis and rule out physiological IGF-1 elevation as a mimic.

Achieving hormonal balance through levothyroxine, hydrocortisone, and fludrocortisone adjustments is complex, as over- or under-replacement can cause glycemic instability, weight changes, and persistent hypertension [14]. The case also highlights acromegaly-associated complications like multinodular goiter, diabetes mellitus, macular degeneration, glaucoma, sleep apnea, and the need for transsphenoidal surgery, which successfully achieved tumor debulking and IGF-1 stabilization without further medical therapy of somatostatin receptor ligands (SRLs), cabergoline, or pegvisomant [15,16].

Cardiovascular disease (CVD) is a frequent comorbidity in acromegaly, driven by GH/IGF-1 hypersecretion, disease duration, and conventional risk factors. Hypertrophic cardiomyopathy, often asymptomatic, may be present at diagnosis and occasionally progresses to heart failure [17]. Additionally, GH and IGF-1 promote cellular proliferation, increasing cancer risk, particularly colonic neoplasia, necessitating regular screening [18,19]. Sleep apnea in acromegaly, primarily obstructive, is caused by airway narrowing from soft tissue changes. These findings underscore the importance of regular, comprehensive follow-up to monitor complications, optimize therapy, and prevent recurrence or disease progression [20].

## Conclusions

This case illustrates the diagnostic and therapeutic complexity of coexisting PAI, post-surgical hypothyroidism, and acromegaly. The diagnosis of acromegaly was confirmed via clinical features, persistent IGF-1 elevation, pituitary imaging, and definitive pathological evidence of a densely granulated GH-secreting adenoma. The overlapping symptoms among the three endocrine conditions significantly delayed diagnosis and required longitudinal reassessment. Our findings highlight the importance of maintaining a high index of suspicion for additional endocrine disorders, even in patients already diagnosed with primary hormonal deficiencies.
